# O-02 - TARGETED METAGENOMIC ANALYSIS OF CAUSES OF INFLUENZA-LIKE AND RESPIRATORY ILLNESS IN HEALTHCARE WORKERS IN THE UK, WINTER 2025/26: THE SIREN PREPARE AND DETECT STUDY

**DOI:** 10.1093/pubmed/fdag046.002

**Published:** 2026-07-09

**Authors:** Ms Sarah Foulkes, Ms Katie Bellis, Ms Marissa Knoll, Ms Katie Munro, Dr Irina Lut, Ms Quisha Bustamante, Mr Jameel Khawam, Mr Jesus Fresno, Ms Angela Dunne, Ms Sophie Russell, Ms Omoyeni Adebiyi, Dr Jasmin Islam, Dr Ana Atti, Dr Ewan Harrison, Dr Victoria Hall

**Affiliations:** UK Health Security Agency; Wellcome Sanger Institute; Wellcome Sanger Institute; UK Health Security Agency; UK Health Security Agency; UK Health Security Agency; UK Health Security Agency; UK Health Security Agency; UK Health Security Agency; UK Health Security Agency; UK Health Security Agency; UK Health Security Agency; UK Health Security Agency; Wellcome Sanger Institute; UK Health Security Agency

## Abstract

**Background:**

Healthcare workers (HCWs) have increased risk of respiratory infections, which contributes to pressures on healthcare systems and makes them a valuable population for surveillance.

**Objectives:**

We used targeted metagenomic sequencing, to understand the range of viruses associated with respiratory illness in HCWs in winter 2025/26.

**Methods:**

A cohort study of HCWs in the United Kingdom, who completed fortnightly symptom and sick-leave surveys and eight weekly nasopharyngeal swabs, December-January. Swabs were categorised as influenza-like illness (ILI), respiratory and asymptomatic based on symptoms. Swabs were selected by symptom category and week for bait capture metagenomic sequencing targeting 29 respiratory viruses based. Swab positivity by symptom category and virus was calculated over time. Symptom severity and demographics were compared by virus detected using logical regression.

**Results:**

655 samples sequenced, from 324 participants. 31% (202/655) samples had a virus detected (41% (54/131) ILI symptoms, 33% (142/425) respiratory, 7% (4/57) asymptomatic) peaking in week 53. Ten different viruses were detected, with rhinovirus (23%), RSV (16%) and influenza A (15%) most frequent. For influenza A positive samples, 74% had ILI symptoms and 26% respiratory, compared with 21% ILI and 79% respiratory for RSV.

No demographic or occupational factors were associated with viral detection, except for being older (>55years: OR1.4; 95%CI0.99-1.9; p-value 0.055). Compared to symptomatic samples with no virus detected, having influenza (5.6; 2.6-11.7; >0.00) or RSV (3.4; 1.6-7.0; 0.001) detected was associated with increased likelihood of taking sick leave.

**Conclusions:**

Multiple respiratory viruses co-circulate in HCWs over winter, and metagenomics has a valuable surveillance role. The presence of respiratory/ILI symptoms in HCWs is strongly associated with viral detection, however ILI is more specific to influenza. These findings emphasise the importance of comprehensive respiratory virus surveillance, targeted infection prevention measures, and workforce planning beyond vaccination to protect healthcare capacity and resilient health services during winter.

